# Tav4SB: integrating tools for analysis of kinetic models of biological systems

**DOI:** 10.1186/1752-0509-6-25

**Published:** 2012-04-05

**Authors:** Mikołaj Rybiński, Michał Lula, Paweł Banasik, Sławomir Lasota, Anna Gambin

**Affiliations:** 1 Institute of Informatics, University of Warsaw, ul. Banacha 2, 02-097, Warsaw, Poland; 2 Mossakowski Medical Research Centre, Polish Academy of Sciences, ul. Pawińskiego 5, 02-106, Warsaw, Poland

## Abstract

**Background:**

Progress in the modeling of biological systems strongly relies on the availability of specialized computer-aided tools. To that end, the Taverna Workbench eases integration of software tools for life science research and provides a common workflow-based framework for computational experiments in Biology.

**Results:**

The Taverna services for Systems Biology (Tav4SB) project provides a set of new Web service operations, which extend the functionality of the Taverna Workbench in a domain of systems biology. Tav4SB operations allow you to perform numerical simulations or model checking of, respectively, deterministic or stochastic semantics of biological models. On top of this functionality, Tav4SB enables the construction of high-level experiments. As an illustration of possibilities offered by our project we apply the multi-parameter sensitivity analysis. To visualize the results of model analysis a flexible plotting operation is provided as well. Tav4SB operations are executed in a simple grid environment, integrating heterogeneous software such as Mathematica, PRISM and SBML ODE Solver. The user guide, contact information, full documentation of available Web service operations, workflows and other additional resources can be found at the Tav4SB project’s Web page: http://bioputer.mimuw.edu.pl/tav4sb/.

**Conclusions:**

The Tav4SB Web service provides a set of integrated tools in the domain for which Web-based applications are still not as widely available as for other areas of computational biology. Moreover, we extend the dedicated hardware base for computationally expensive task of simulating cellular models. Finally, we promote the standardization of models and experiments as well as accessibility and usability of remote services.

## Background

The Taverna Workbench [1] is a tool which facilitates the design and execution of *in silico* experiments. The experiments are constructed as workflows which can be stored and executed when needed. The building blocks of a workflow are services, also known as processors. Technically, workflow is a set of processors, together with connections between their inputs and outputs. The remote processors are implemented as Web service (WS) [2] operations. Scattered physically throughout computational resources of numerous scientific facilities and combined together, the WSs operations enable a highly complex analysis, surpassing limits of a common workstation.

Taverna services come from a diverse set of life science domains. In the field of computational biology, the Taverna Workbench provides an access to services which are mainly related to the sequence annotation and analysis. Here, we present remote processors that extend Taverna’s functionality in the domain of systems biology, specifically, in the analysis of kinetic models of biological systems. Our hardware base offers computational resources sufficient for computationally demanding experiments, such as multiple invocations of the model-checking procedure. Essentially, the Taverna Workbench provides a convenient user interface for our WS operations. Without programming their own WS client, users can analyze the behavior of cellular systems under various conditions.

## Features

For a given biochemical network model, the underlying mathematical model is determined by the chosen semantics. The most common representations are ordinary differential equations (ODEs) for the deterministic framework and continuous-time Markov chain (CTMC) for the framework [3,4]. The latter representation may be equivalently expressed as a set of differential equations, know also as the chemical master equation. Unlike the Tav4SB project, almost all of the Web-based applications reviewed in [5] allow for the analysis of only deterministic representations of biological systems.

Operations provided by our Web server allow for:

1. numerical simulations for the deterministic formulation of a biochemical network model, using the SBML ODE Solver library (SOSlib) [6],

2. probabilistic model checking of Continuous Stochastic Logic (CSL) [7] formula over a CTMC, using PRISM [8],

3. visualization of data series, such as ODEs trajectories or values of parametrized CSL properties, and probabilistic distribution sampling, using Mathematica [9], and

4. high-level analysis, such as multi-parameter sensitivity analysis (MPSA) [10] of biological models, with error calculation via either numerical simulations or the probabilistic model checking technique.

The SBML ODE Solver library enables numerical analysis of models encoded directly in Systems Biology Markup Language (SBML) [11]. The library employs libSBML [12] to automatically derive ODEs, plus their Jacobian and higher derivatives, as well as the CVODES package — the state of the art numerical integration library from SUNDIALS [13].

PRISM is one of the leading tools implementing probabilistic model checking, a technique of formal verification of systems that exhibit a stochastic behavior. A system to be analyzed is modeled as a Markov chain, and an examined property is expressed in a suitable probabilistic temporal logic. Some recent works, see e.g. [14,15], demonstrate applicability of PRISM to analysis of models of biological systems. Case studies include models of cell cycle control, fibroblast growth factor signaling, and MAPK cascade [16]. For biological applications a CTMC is typically chosen as an underlying mathematical model and its properties are specified in a continuous time logic, for instance in CSL. This approach seems promising and, compared with numerical simulations, it can often yield a better understanding of the dynamics of analyzed systems.

PRISM handles models defined in the PRISM input language. Currently, a prototype translator from SBML is not integrated into the application itself. Therefore, we also provided a separate operation to automatically translate from SBML to the PRISM language, using the prototype translator.

Finally, Wolfram’s Mathematica is a tool with one of the most advanced graphics engines among plotting software. Tav4SB provides Mathematica’s two- and three-dimensional list plots together with a versatile set of options for customizing their display. Additionally, Tav4SB allows to sample from the extensive collection of parametric probability distributions available in Mathematica.

## Context

The aim of the Tav4SB project is to support the orchestration of physically scattered tools for execution of repeatable scientific experiments To understand a place of Tav4SB in a plethora of similar software, consider the following, mundane technical problem. You have a set of scripts, command line tools or any other form of legacy code, installed on one or more computational servers, not necessarily in the same local area network. For instance, you might have a Mathematica script which can be only executed on a server which has Mathematica installed on it; and simultaneously you might need to use PRISM, installed on a remote server with a large amount of required memory. You want to connect these tools in an *in silico* experiment, say described by a workflow. Moreover, in case the experiment doesn’t go as planned, you want to be able to easily modify and re-run your workflow.

Tav4SB project is a realization of a minimalist approach to a platform-independent solution, based on the workflow management system and a service-oriented architecture built around the Web service standard and a straightforward queue of computational tasks.

Tav4SB project consists of two parts. The client part of the project (Tav4SB client) is a library of sample workflows and helper scripts for analysis of kinetic models of biological systems, using earlier described features. The server part of the project (Tav4SB server) is a simple grid environment which wraps aforementioned computational tools. Those tools are intended to be run in a multi-threaded manner, on one or more, possibly remote, computational servers.

As an utility for wrapping scientific software in Web services, the Tav4SB project enters premises of projects such as Soaplab2 [17] and Opal2 [18,19]. The main difference is that the support for the physical scattering of computational tools is an integral part of the Tav4SB server. Moreover, Tav4SB server easily allows for a direct connection with legacy code. If necessary, the Java Native Interface (JNI) [20] can be used to connect with the platform-specific libraries written, for instance in C, C++, or Fortran. However, in the current state of the project, all that comes at a cost of moderate programming skills required from a user of the Tav4SB server, when compared to Soaplab2 and Opal2 strategy with the custom configuration file languages. Please note however that these languages need to be learned and they pose an easier approach for the user only to a limited extend. Also note that, as a minimalist solution with the stateless Web service interface, the Tav4SB server doesn’t comply with the standards of an open, stateful grid services architecture (cf. Web Services Resource Framework [21]), which the most prominent representative is Globus Toolkit [22], a full-fledged grid environment.

## Implementation

We have chosen the popular Systems Biology Markup Language (SBML) [11], an XML-based data format, to represent kinetic models of biological systems. Due to the wide range of dedicated software and due to the support by models repositories like BioModels [23], SBML can be used without a detailed knowledge of the language specification.

**Figure 1:**
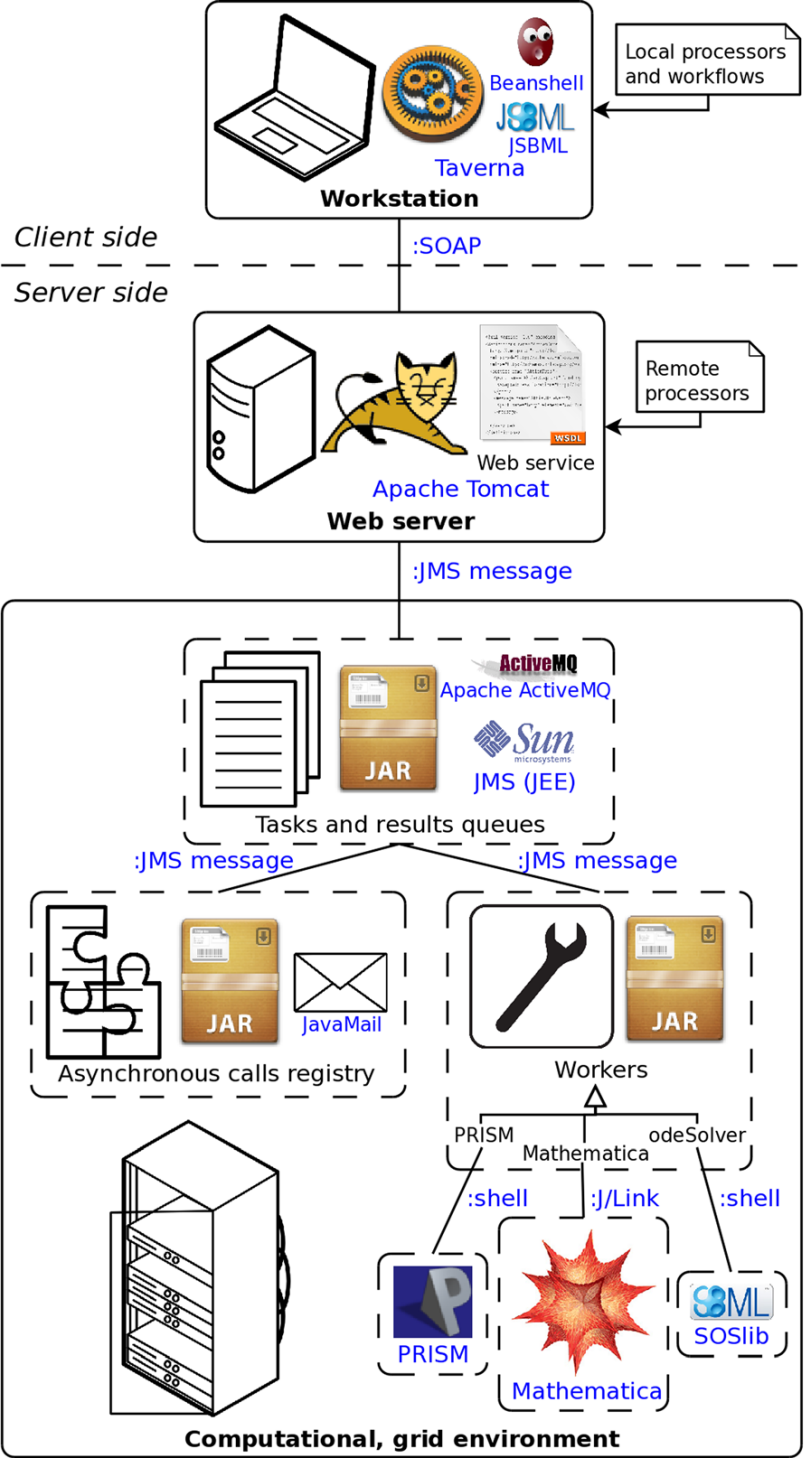
**The implementation architecture.** Names of a particular software, technology or standard are written in blue. The communication type is specified on edges which connect components of the system. See text for details.

Figure 1 depicts the architecture of our solution. The client side includes a workstation with the Taverna Workbench installed. Besides remote processors, the Taverna Workbench provides access to local processors. These might be locally-installed command-line programs, including environments for running scripts, which enable data manipulation on the client side. Moreover, scripts written in BeanShell — an interpreter for a simplified version of Java language, are natively supported by the Taverna Workbench, constituting a highly portable workflow design. Thanks to local processors, lightweight computations can be executed on a user’s machine. This makes workflows more effective by reducing network load, response time and the burden on the server side. To that end, we used a native Java SBML library (JSBML) [24]. JSBML enables client side manipulation of the SBML models, for instance to extract parameter names from a model and to set their values.

Client communicates with the server side via WS operations, using Simple Object Access Protocol (SOAP) [25]. These operations represent the workflow’s remote processors. Their signatures are defined in a Web Service Definition Language (WSDL) [26] file. We employed a “WSDL first” approach: the WSDL file was manually written (in a document/literal style).

Java Web service classes were automatically generated from the WSDL file.

The WSDL file is hosted by the Apache Tomcat servlet container. It acts as a proxy between the client and the computational part of the server. A Web service operation call is translated into a Java Message Service (JMS) [27] messages. JMS Application Programming Interface (API) allows Java applications to create, send, receive, and read messages. It is a part of the Java Platform, Enterprise Edition (JEE) standards. In our system, JMS messages represent computational tasks, and their results. One operation call can be translated into multiple tasks, enabling seamless, tool-specific parallelization of a submitted job.

Computational cluster management modules are written in Java using the Apache ActiveMQ implementation of the JMS standard. These modules are deployed as the Java Archive (JAR) files. The JMS messages are sent over TCP/IP, which basically makes modules independent of their physical location.

New tasks, created by the Web server module, are added to the tasks queue. At this point tasks are assigned to any available worker of a compatible type. Results are collected in a temporary queue, exclusive for a single WS operation call. Long-running tasks use an asynchronous call registry. In such case, direct (synchronous) response to the WS operation call is merely a message reporting the start of computations. The computed results are collected in a dedicated queue and, when completed, sent to a caller by email (using the JavaMail package).

Worker translates both a JMS task message into running computational processes and results of these processes back into a JMS result message. Each worker supports a specific type of computation and can communicate with an actual computational tool differently. Currently we implemented three types of workers: Mathematica worker which communicates with Mathematica via J/Link library, PRISM and odeSolver workers which communicate with, respectively, PRISM and SOSlib via a command-line interpreter (shell).

## Results and Discussion

We constructed a set of exemplary workflows. Their main purpose is to demonstrate how Tav4SB WS operations can be used by the Taverna Workbench client. There are two kinds of workflows: Tav4SB WS operation wrappers and *in silico* experiments.

Wrapper workflows illustrate a direct usage of Tav4SB operations in Taverna. Their purpose is to be re-used as nested workflows — building blocks of experiments described below. Additionally, we built a number of helper Taverna processors, used for interacting with XML-formatted inputs and outputs of WS operations.

In all our *in silico* experiments we have used the enzymatic reaction model:

(1) $$E+S\overset{k_{1}}{\underset{\underset{k_{2}}{↽}}{⇀}}ES\overset{k_{3}}{\to }E+P$$

The species names S, E, ES and P stand for substrate, enzyme, enzyme-substrate complex and product, respectively. Length of an arrow indicates the order of the reaction rate. Initial amounts of species and kinetic parameters values, taken from [28], are

(2) $$\begin{array}{c} {\text{S}}_{0}=12\;{\text{E}}_{0}=10\;{\text{ES}}_{0}=0\;{\text{P}}_{0}=0,\\ k_{1}=0.184\;k_{2}=0.016\;k_{3}=\text{0.211.} \end{array}$$

## Numerical ODEs simulations

The first workflow numerically simulates the ODEs of the model and plots resulting trajectories. ODEs are derived automatically from a SBML model file, based on rate laws of reactions. In the deterministic model of the enzymatic reaction, rates are described by the law of mass-action. As a result of running this simple experiment one gets time evolution of species concentrations in the form of both data points series and a plot.

**Figure 2:**
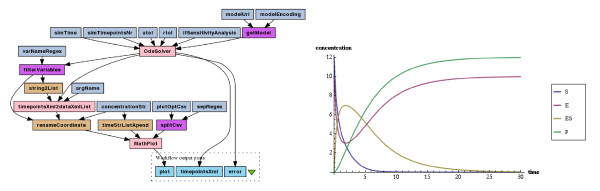
**The “Simulate SBML-derived ODEs” workflow and resulting trajectories plot for the enzymatic reaction model (Equations (1) and (2)).** Pink boxes represent nested workflows, corresponding to Tav4SB WS operations wrappers and a helper. See text for more details.

Figure 2 depicts the simulation workflow and a resulting plot for all species of the model over a time period of 30 seconds. The system stabilizes in approximately 25 seconds with a peak activity at the 2-nd second. At that time point most of the enzymes are at work, i.e. they are bound to substrates, which, in turn, are converted into the product.

## Probabilistic model checking

The second experiment uses the probabilistic model checking technique to calculate the probability of a property to be satisfied, over a stochastic model of the enzymatic reaction (Equation (1)). The stochastic version is also encoded in the SBML format. The property being checked is expressed as the following reward-based CSL formula:

(3) $$R_{\left\{\#r1=?\right\}}\;\left[◊,\left(P>0.5\cdot \underset{t\to \infty }{lim}P(t)\right)\right]\text{.}$$

**Figure 3:**
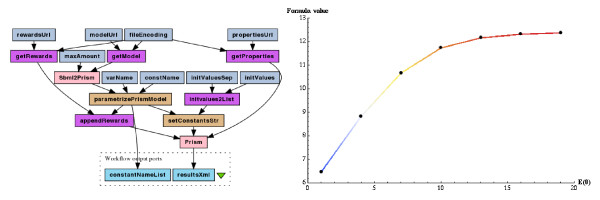
**The computational part of the “Probabilistic model checking of the SBML stochastic model” workflow and the resulting plot for the stochastic model of the enzymatic reaction (Equation (1)).** Pink boxes represent nested workflows, corresponding to Tav4SB WS operations wrappers. See text for more details.

Roughly speaking, this formula answers the following question: how many times, on average, the reaction *r1* of association of the enzyme-substrate complex has to occur, before the amount of the product *P* reaches 50% of its maximum? It is motivated by the half maximal effective concentration (*EC*_50_ coefficient). The formula is evaluated for different enzyme initial amounts to find the enzyme’s optimal efficiency. As this is not an instantaneous computation and plotting usually requires many repeats to fine-tune a plot parameters, the experiment is divided into two separate parts: a computational part and a plotting part. Figure 3 depicts the computational part of the workflow and the resulting plot. The plot can be read as follows: if *E(0)* is equal to 1 then, on average, before the product reaches half of its maximum, each enzyme has to convert slightly more than 6 substrates. When *E(0)* is equal to 12 (the initial amount of the substrate), each enzyme converts on average at most one substrate. The total, parallel efficiency of the enzymatic reaction model doesn’t improve significantly from that point on. Not much more than 12 complex formation reactions *r1* are needed to achieve half of the maximum product amount.

## Multi-parameter sensitivity analysis

Sensitivity analysis investigates a relation between uncertain input or parameters of a model, and a property of an observable output [10,29]. Sensitivity analysis has been used for various parametrization tasks of models of biological systems, including finding essential parameters for research prioritization [30], identifying insignificant parameters for the model reduction [31] or parameters clustering for the discovery of common functions [32].

Biochemical reaction networks yield models of a nonlinear nature for which global sensitivity analysis methods (GSA) are the most suitable [29]. GSA examines a range of input parameter values simultaneously as opposed to one-factor-at-a-time methods such as those calculating the derivatives of output with respect to parameters. Multi-parameter sensitivity analysis (MPSA) [33] is an implementation of the GSA concept. MPSA is an instance of a Monte Carlo filtering method, which maps samples from a parameter space into behavioral and non behavioral output regions [10]. For the examples of applications of the multi-parameter sensitivity analysis to signaling pathways see [28,34]. The MPSA method works as follows:

1. Select parameters to assess.

2. Set parameters range.

3. Generate independent samples.

4. For each sample calculate the error (based on the output).

5. Classify samples as acceptable or unacceptable.

6. For each of the selected parameters compare the classified samples sets.

**Figure 4:**
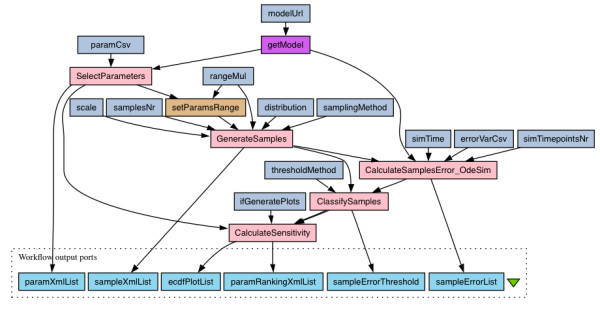
**The multi-parameter sensitivity analysis workflow with an ODE-based error function.** Pink and brown boxes represent essential steps of the procedure. Remaining boxes represent workflow’s parameters and outputs. See text for details.

This procedure is depicted in Figure 4 as a workflow.

Calculating the error for each sample (Step 4) involves a separate analysis of the model. This is a factor that determines the running time of the MPSA procedure. We ran two variants of MPSA, differing in the way in which the error is calculated. In one variant we used ODEs simulations and in the other one we exploited the probabilistic model checking technique. We focused on kinetic parameters of two forward reactions of enzymatic reaction models (Equation (1)), i.e. *k*_1_ and *k*_3_. As an error function we took, respectively, the mean squared error of an ODE trajectory of the product P and the absolute difference of the value of the formula (3), in both cases between results for a parameters sample and for the reference values of parameters (Equation (2)). In turn, we obtained empirical cumulative distribution functions (ECDF) of acceptable and unacceptable samples, for each of the selected parameters. ECDFs were compared using the Kolmogorov-Smirnov test (KS-test) and one minus the Pearson product–moment correlation coefficient (PMCC). As a final output of the MPSA method, we got two rankings for each of the sensitivity indices: KS-test and PMCC.

**Figure 5:**
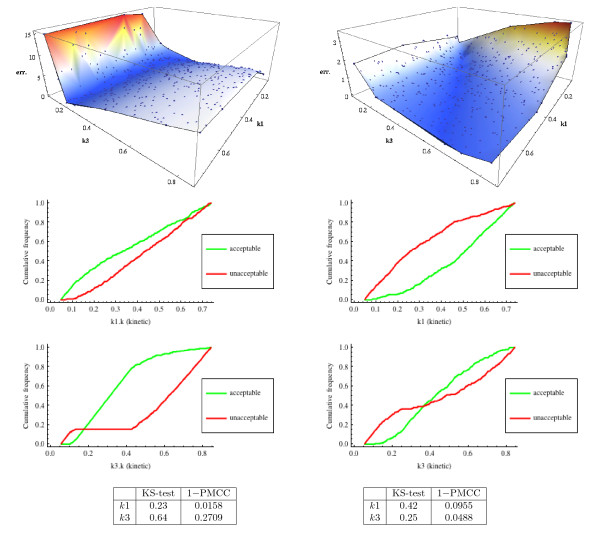
**MPSA error surfaces, ECDFs and values of sensitivity indices for error calculated using deterministic model with the mean squared error of product trajectories (left column) and using stochastic model with the absolute difference of a value of the formula (3) (right column).** Both procedures were run for 400 samples of parameters. Samples were generated using the Latin hypercube sampling method [36], from a uniform distribution over a range from $\frac{1}{4}\times$ to 4 × the nominal value of each of investigated parameters (Equation (2)). As a threshold for classifying samples as acceptable or unacceptable, we took median of error values. The larger the value of a statistics which compares ECDFs, the more significant is a parameter with respect to a property of interest.

Figure 5 depicts values of the error function and ECDFs of acceptable and unacceptable samples, for parameters *k*_1_ and *k*_3_, for both variants of the MPSA procedure. In the variant based on ODEs simulations and the error function which measures changes in the product *P* trajectory, one clearly observes that parameter *k*_3_ significantly dominates parameter *k*_1_, as far as sensitivity of the system is concerned. This is an expected result. Firstly, *k*_3_ is a rate parameter of a reaction which is directly responsible for a product creation. Secondly, from the Michaelis-Menten approximation [35]:

$$\frac{d[\text{P}](t)}{dt}\approx k_{3}[\text{E}](0)\frac{\left[\text{S}](t\right)}{[\text{S}](t)+\frac{k_{2}+k_{3}}{k_{1}}}\text{ ,}$$

one can expect that, for values from Equation (2), variation of parameter *k*_3_ will be more influential, with respect to the product rate, than variation of parameter *k*_1_.

Interestingly, the results of the other variant of the MPSA procedure are significantly different; one observes that now *k*_1_ dominates *k*_3_. This may be ascribed to the particular choice of the formula (3) which calculates the average number of occurrences of the first reaction *r*_1_. Furthermore, an inspection of values of sensitivity indices given in Figure 5 brings to light that the domination is not as definite as in the first variant of MPSA. Results demonstrate that an application of the probabilistic model checking technique may allow for revealing more subtle dependencies in the model, depending on the properties of interest.

MPSA combined with PMC may be applied as a pre-processing step which finds parameters that are insignificant for an analysis oriented on a very specific property of a model. This would provide a novel notion of a probabilistic abstraction [37], i.e. property-specific reduction of the probabilistic model. However, for a successful application, the pre-processing should have low running time, compared to an analysis that follows. In our experiment this is not the case, as we run the exact PMC procedure, which is essentially the same one that would be ran during the further analysis. However, we conjecture that for the MPSA procedure the level of accuracy offered by PRISM is much too high. We suppose that satisfactory results may be obtained using an approximate approach, such as Monte Carlo model-checking [38]. We plan to pursue this idea as a continuation of the work presented here.

## Performance test

To measure the network load and the overhead of the task management in Tav4SB server we ran a performance test. The test was set up with the MAPK cascade case study from the PRISM Web page [16] and with the asynchronous version of the PRISM WS operation. This version of the PRISM operation sends computation time statistics, together with results (by email). To run the performance test, we deployed the Tav4SB server on the conventional, computational cluster maintained by the Center of Excellence BioExploratorium at the University of Warsaw. The cluster contains 16 machines with 2 dual-core CPUs each, giving 64 cores in total. We used 14 machines to deploy workers and 1 machine for the management queue. The Web server was deployed on a separate gate server.

**Figure 6:**
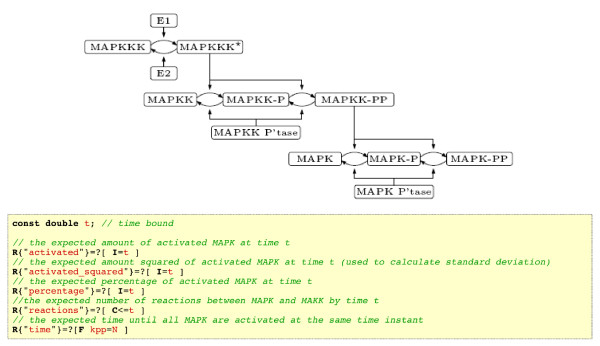
**A scheme of the MAPK cascade model and a list of verified properties from the PRISM Web page **[16].

The stochastic MAPK model (see Figure 6) defines the level of granularity of the represented system, denoted by *N*. *N* is the maximal number of molecules for each of the model species. Size the an underlying CTMC grows exponentially with the granularity. Additionally, verified properties (see Figure 6) depend on a time point parameter *t*. The longest model checking task, for a fixed *t* value, took just over 9 minutes for *N* = 3, approximately half a minute for *N* = 2 and just few seconds for *N* = 1. We repeated the test multiple times, each time for *N* = 1, 2, 3 and *t* = 1, …, 50, i.e. total of 150 PRISM tasks; 50 fast, 50 medium and 50 long running computations.

Table 1 contains the average longest computation time with varying numbers of machines and numbers of threads for each worker on each machine. With a separate core for each worker and with enough workers to cover long running computations (4 threads on 14 machines) one can observe an effect of running a single time point simulation. Tav4SB server scales well in a local, homogeneous environment. There is only a small overhead which may be attributed to the worker initialization, the task management and the network load. Also note that in a situation of a sufficient number of workers, running more threads than the available number of cores (8 threads on 8 and 14 machines), the environment significantly slows down. It happens because workers fight for the processor time, causing the operating system to frequently switch context of a current processor task. On the other hand, in a situation of high deficiency of workers (1, 2 or 4 machines), with 100 computations running in 30 seconds or less, it is better to have more threads than cores on each machine (8 threads). This results mainly from an overhead of the tasks queuing, initialization of workers the communication between grid components. To sum up, for the optimal production deployment, one must consider the amount and the time profile of tasks which are being executed with respect to the thread per core ratio which must be adjusted accordingly; basically, high ratio for many short tasks and inversely for long running tasks.

**Table 1:** Results of the performance test of Tav4SB server

**# of threads/machines**	**1**	**2**	**4**	**8**	**14**
**1**	271,25	137,84	71,22	38,06	23,85
**2**	149,09	71,12	38,00	21,51	14,55
**4**	124,53	54,37	26,06	13,68	9,75
**8**	70,44	37,53	23,78	17,39	16,44

Table cells contain the average longest computation time in minutes, in different configurations of a number of machines and a number of threads for each worker.

## Conclusions

Web-based applications are still not as widely available for the systems biology domain as for other research areas [5]. One reason for this state of affairs is the fact that simulating cellular models is computationally expensive, when compared to the data processing tasks. In turn, there is a constant demand for a hardware dedicated to the analysis of kinetic models of biological systems [5].

Our services extend the functionality of the Taverna Workbench in the field of systems biology. Together with the services we provide a hardware base for our minimalist grid environment. The grid itself can, and will be, easily extended, independently of a physical location of peripherals and independently of an operating system they are running. Moreover, our grid facilitates integration of heterogeneous tools, such as Mathematica, PRISM or SOSlib. The end-user goal of the Tav4SB project is to abstract details of the technological infrastructure. Finally, via SBML and the Taverna Workbench, we would like to promote standardization of models and experiments as well as accessibility of services and their usability for non-programmers. In order to further enhance the usability, we released the source code of the project so that users can extended the Tav4SB functionality with their own workers modules. Users with programming skills can contribute to the development of the technical aspects of the server part of the project. These aspects cover the plug-in architecture of workers, the library of legacy code connectors (e.g., currently used, command-line interface or Java library), descriptors for the automatic generation of the workers code for common types of wrapped applications (cf. ACD metadata files in the Soaplab2 project [17]), and, last but not least, the support for Semantic Web services and ontologies [39-41].

From the point of view of *in silico* experiments, we propose a novel technique: application of the probabilistic model checking to the calculation of error in the multi-parameter sensitivity analysis procedure. It seem that this approach is particularly well suited for revealing intricate and subtle dependencies, that may not be discovered using, for instance, ODE-based numerical simulations of a model. We suppose that this technique may have interesting applications, e.g. for probabilistic abstraction [37].

## Availability and requirements

• Project name: Tav4SB

• Project home page: http://bioputer.mimuw.edu.pl/tav4sb/

• Operating system(s): Platform independent (both client and server parts)

• Programming language: Optionally, SCUFL/t2flow, BeanShell, XSLT (client) and Java, Mathematica, Bash (server)

• Other requirements: the Taverna Workbench client 2.3 or higher, JSBML 0.8-b2, plus, optionally, any files hosting Web server (client) and Apache Tomcat 6.0 series, Apache Maven 2 or higher, plus, optionally, Mathematica 7.0 or higher, PRISM 4.0 series and SBML ODE Solver 1.6 (server)

• License: GNU AGPL

• Any restrictions to use by non-academics: None

Please note that, technically, SCUFL and t2flow are workflow description languages, but together with the graphical notation provided by the Taverna Workbench they can be seen as visual programming languages. These and other client dependencies on a programming language are optional because one can write their own WS client in virtually any language. Also, be advised that the Apache Maven tool (in other requirements) automatically resolves all dependencies on Java libraries, such as JavaMail or Apache ActiveMQ (cf. Figure 1).

The definition of operations provided by Tav4SB WS plus workflows files, together with installation and execution instructions are available from the project’s home page. Documentation of the Tav4SB WS can be found in BioCatalogue [39], a curated catalogue of life sciences Web services. Wrappers and experiments workflows are also available from the myExperiment repository [42], together with the workflow figures.

Client workflows were tested on Ubuntu Linux (10.10), Mac OS X (10.6.8) and Windows Vista (Business) operating systems. The production server is currently deployed on computational servers at the Faculty of Mathematics, Informatics and Mechanics of the University of Warsaw (running Ubuntu Linux Server, Gentoo Linux and PLD Linux). The performance test server was deployed on a cluster of Ubuntu Linux machines (workers and queue) and Solaris gateway (WS). A local developer’s environment, with both client and server, was deployed and tested on Ubuntu Linux (10.10) and Mac OS X (10.6.8).

## Competing interests

The authors declare that they have no competing interests.

## Authors’ contributions

MR performed experimental parts, implemented the system and prepared the final version of the manuscript. ML implemented the grid structure of the system and performed performance tests. PB did the initial implementation. AG and SL supervised this project and participated in drafting the manuscript. All authors have read and approved the final manuscript.

## References

[B1] Hull D Wolstencroft K Stevens R Goble C Pocock MR Li P Oinn T Taverna: a tool for building and running workflows of services Nucleic Acids Research 2006 34 729 732 10.1093/nar/gkl320 PMC1538887 16845108

[B2] The World Wide Web Consortium: Web Services Activity [http://www.w3.org/2002/ws/] (Last accessed: 11 January 2012).

[B3] Aldridge BB Burke JM Lauffenburger DA Sorger PK Physicochemical modelling of cell signalling pathways Nature Cell Biology 2006 8 11 1195 1203 10.1038/ncb1497 17060902

[B4] Wolkenhauer O Ullah M Kolch W Cho K Modeling and simulation of intracellular dynamics: choosing an appropriate framework IEEE Transactions on Nanobioscience 2004 3 3 200 207 10.1109/TNB.2004.833694 15473072

[B5] Lee D Saha R Yusufi FNK Park W Karimi IA Web-based applications for building, managing and analysing kinetic models of biological systems Briefings in Bioinformatics 2009 10 65 74 18805901 10.1093/bib/bbn039 PMC2638623

[B6] Machné R Finney A Müller S Lu J Widder S Flamm C The SBML ODE Solver Library: a native API. for symbolic and fast numerical analysis of reaction networks Bioinformatics 2006 22 1406 1407 10.1093/bioinformatics/btl086 16527832

[B7] Aziz A Sanwal K Singhal V Brayton R Verifying continuous time Markov chains 1996 Springer: In Proc. 8th International Conference on Computer Aided Verification (CAV’96), Volume 1102 of Lecture Notes in Computer Science 269 276

[B8] Hinton A Kwiatkowska M Norman G Parker D: PRISM A Tool for Automatic Verification of Probabilistic Systems 2006 Springer: In Proc. 12th International Conference on Tools and Algorithms for the Construction and Analysis of Systems (TACAS’06), Volume 3920 of Lecture Notes in Computer Science 441 444

[B9] Wolfram Research, Inc Mathematica Edition 2008 Champaign, Illinois: Version 7.0

[B10] Saltelli A Ratto M Andres T Campolongo F Cariboni J Gatelli D Saisana M Tarantola S Global Sensitivity Analysis 2008 Wiley-Interscience: The Primer

[B11] Hucka M Finney A Sauro HM Bolouri H Doyle JC Kitano H Arkin AP Bornstein BJ Bray D Cornish-Bowden A Cuellar AA Dronov S Gilles ED Ginkel M Gor V Goryanin II Hedley WJ Hodgman TC Hofmeyr JH Hunter PJ Juty NS Kasberger JL Kremling A Kummer U Le Novère N Loew LM Lucio D Mendes P Minch E Mjolsness ED Nakayama Y Nelson MR Nielsen PF Sakurada T Schaff JC Shapiro BE Shimizu TS Spence HD Stelling J Takahashi K Tomita M Wagner J Wang J The systems biology markup language (SBML): a medium for representation and exchange of biochemical network models Bioinformatics 2003 19 524 531 10.1093/bioinformatics/btg015 12611808

[B12] Bornstein BJ Keating SM Jouraku A Hucka M LibSBML: an API library for SBML Bioinformatics 2008 24 6 880 1 10.1093/bioinformatics/btn051 18252737 PMC2517632

[B13] Hindmarsh AC Brown PN Grant KE Lee SL Serban R Shumaker DE Woodward CS SUNDIALS: Suite of nonlinear and differential/algebraic equation solvers ACM Transactions on Mathematical Software 2005 31 3 363 396 10.1145/1089014.1089020

[B14] Heath J Kwiatkowska M Norman G Parker D Tymchyshyn O Probabilistic model checking of complex biological pathways Theoretical Computer Science 2008 391 3 239 257 10.1016/j.tcs.2007.11.013

[B15] Kwiatkowska M Norman G Parker D Using probabilistic model checking in systems biology ACM SIGMETRICS Performance Evaluation Review 2008 35 4 14 21 10.1145/1364644.1364651

[B16] PRISM Web page 2012 [http://www.prismmodelchecker.org/] (Last accessed: 11 January 2012).

[B17] Senger M Rice P Bleasby A Oinn T Uludag M Soaplab2: more reliable Sesame door to bioinformatics programs 2008 Toronto, Canada: In The 9th Annual Bioinformatics Open Source Conference

[B18] Krishnan S, Clementi L, Ren J, Papadopoulos P, Li W: Design and Evaluation of Opal2: A Toolkit for Scientific Software as a Service. In Proceedings of the Congress on Services - I Washington, DC, USA: IEEE Computer Society 2009 2009 709 716

[B19] Ren J Williams N Clementi L Krishnan S Li WW Opal web services for biomedical applications Nucleic Acids Research 2010 38 724 731 10.1093/nar/gkq503 PMC2896135 20529877

[B20] Oracle Corporation Java™ Native Interface 2012 [http://docs.oracle.com/javase/7/docs/technotes/guides/jni/] (Last accessed: 16 January 2012).

[B21] OASIS WSRF Technical Committee OASIS Web Services Resource Framework (WSRF) version 1.2 standard 2012 [http://www.oasis-open.org/committees/wsrf/] (Last accessed: 16 January 2012).

[B22] Foster I Globus Toolkit Version 4: Software for Service-Oriented Systems Journal of Computer Science and Technology 2006 21 4 513 520 10.1007/s11390-006-0513-y

[B23] Le Novère N Bornstein B Broicher A Courtot M Donizelli M Dharuri H Li L Sauro H Schilstra M Shapiro B Snoep JL Hucka M BioModels Database: a free, centralized database of curated, published, quantitative kinetic models of biochemical and cellular systems Nucleic Acids Research 2006 34 Database issue 689 91 10.1093/nar/gkj092 PMC1347454 16381960

[B24] DrÃ¤ger A Rodriguez N Dumousseau M Dörr A Wrzodek C Le Novère N Zell A Hucka M JSBML: a flexible Java library for working with SBML Bioinformatics (Oxford, England) 2011 27 15 2167 2168 10.1093/bioinformatics/btr361 PMC3137227 21697129

[B25] The World Wide Web Consortium SOAP version 1.2 2012 [http://www.w3.org/TR/soap12/] (Last accessed: 11 January 2012).

[B26] Christensen E Curbera F Meredith G Weerawarana S Web Service Definition Language (WSDL) 2001 [http://www.w3.org/TR/wsdl] (Last accessed: 11 January 2012).

[B27] Hapner M Burridg R Sharma R Fialli J Stout K Java Message Service 1, Sun Microsystems, Inc: Specification, version: 1

[B28] Cho KH Shin SY Kolch W Wolkenhauer O Experimental Design in Systems Biology, Based on Parameter Sensitivity Analysis Using a Monte Carlo Method: A Case Study for the TNFα–Mediated NF–κB Signal Transduction Pathway SIMULATION 2003 79 726 739 10.1177/0037549703040943

[B29] Saltelli A Ratto M Tarantola S Campolongo F Sensitivity analysis for chemical models Chemical Reviews 2005 105 2811 28 10.1021/cr040659d 16011325

[B30] Yue H Brown M He F Jia J Kell D Sensitivity analysis and robust experimental design of a signal transduction pathway system International Journal of Chemical Kinetics 2008 40 11 730 741 10.1002/kin.20369

[B31] Shankaran H Wiley HS Resat H Modeling the effects of HER/ErbB1-3 coexpression on receptor dimerization and biological response Biophysical Journal 2006 90 11 3993 4009 10.1529/biophysj.105.080580 16533841 PMC1459488

[B32] Mahdavi A Davey RE Bhola P Yin T Zandstra PW Sensitivity Analysis of Intracellular Signaling Pathway Kinetics Predicts Targets for Stem Cell Fate Control PLoS Computational Biology 2007 3 e130 10.1371/journal.pcbi.0030130 17616983 PMC1913098

[B33] Hornberger GM Spear RC An approach to the preliminary analysis of environmental systems Journal of Environmental Management 1981 12 7 18

[B34] Zi Z Cho KH Sung MH Xia X Zheng J Sun Z In silico identification of the key components and steps in IFN–gamma induced JAK–STAT. signaling pathway FEBS Letters 2005 579 1101 1108 10.1016/j.febslet.2005.01.009 15710397

[B35] Michaelis L Menten M Die Kinetik der Invertinwirkung. Biochemische Zeitschrift 1913 49 333 369

[B36] McKay M Conover W Beckman R A Comparison of Three Methods for Selecting Values of Input Variables in the Analysis of Output from a Computer Code Technometrics 1979 21 2 239 245

[B37] Laplante S Lassaigne R Magniez F Peyronnet S de Rougemont M Probabilistic abstraction for model checking: An approach based on property testing ACM Transactions on Computational Logic 2007 8 4

[B38] Grosu R Smolka SA Monte Carlo Model Checking 2005 Springer: In Proc. 11th International Conference on Tools and Algorithms for the Construction and Analysis of Systems (TACAS’05), Volume 3440 of Lecture Notes in Computer Science 271 286

[B39] Bhagat J Tanoh F Nzuobontane E Laurent T Orlowski J Roos M Wolstencroft K Aleksejevs S Stevens R Pettifer S Lopez R Goble CA BioCatalogue: a universal catalogue of web services for the life sciences Nucleic Acids Research 2010 38 Web Server issue 689 694 10.1093/nar/gkq394 PMC2896129 20484378

[B40] Wilkinson MD Senger M Kawas E Bruskiewich R Gouzy J Noirot C Bardou P Ng A Haase D Saiz EA Wang D Gibbons F Gordon PMK Sensen CW Carrasco JMR FernÃ¡ndez JM Shen L Links M Ng M Opushneva N Neerincx PBT Leunissen JAM Ernst R Twigger S Usadel B Good B Wong Y Stein L Crosby W Karlsson J Royo R PÃ¡rraga I RamÃ­rez S Gelpi JL Trelles O Pisano DG Jimenez N Kerhornou A Rosset R Zamacola L Tarraga J Huerta-Cepas J Carazo JM Dopazo J Guigo R Navarro A Orozco M Valencia A Claros MG PÃ©rez AJ Aldana J Rojano MM Fernandez-Santa Cruz R Navas I Schiltz G Farmer A Gessler D Schoof H Groscurth A Interoperability with Moby 1.0–it’s better than sharing your toothbrush! Briefings in Bioinformatics 2008 9 3 220 231 18238804 10.1093/bib/bbn003

[B41] Courtot M Juty N KnÃ¼pfer C Waltemath D Zhukova A DrÃ¤ger A Dumontier M Finney A Golebiewski M Hastings J Hoops S Keating S Kell DB Kerrien S Lawson J Lister A Lu J Machne R Mendes P Pocock M Rodriguez N Villeger A Wilkinson DJ Wimalaratne S Laibe C Hucka M Le NovÃ¨re N Controlled vocabularies and semantics in systems biology Molecular Systems Biology 2011 7 543 22027554 10.1038/msb.2011.77 PMC3261705

[B42] Goble CA Bhagat J Aleksejevs S Cruickshank D Michaelides D Newman D Borkum M Bechhofer S Roos M Li P Roure DD myExperiment: a repository and social network for the sharing of bioinformatics workflows Nucleic Acids Research 2010 38 Web Server issue 677 682 10.1093/nar/gkq429 PMC2896080 20501605

